# Comparative analysis of chloroplast genomes reveals phylogenetic relationships and intraspecific variation in the medicinal plant *Isodon rubescens*

**DOI:** 10.1371/journal.pone.0266546

**Published:** 2022-04-06

**Authors:** Conglong Lian, Hao Yang, Jinxu Lan, Xueyu Zhang, Fei Zhang, Jingfan Yang, Suiqing Chen

**Affiliations:** School of Pharmacy, Henan Key Laboratory of Chinese Medicine Resources and Chinese Medicine Chemistry, Henan University of Traditional Chinese Medicine, Jinshui District, Zhengzhou, China; International Centre for Genetic Engineering and Biotechnology, INDIA

## Abstract

*Isodon rubescens* (Hemsley) H. Hara (Lamiaceae) is a traditional Chinese medicine plant that has been used to treat various human diseases and conditions such as inflammation, respiratory and gastrointestinal bacterial infections, and malignant tumors. However, the contents of the main active components of *I*. *rubescens* from different origins differ significantly, which greatly affected its quality. Therefore, a molecular method to identify and classify *I*. *rubescens* is needed. Here, we report the DNA sequence of the chloroplast genome of *I*. *rubescens* collected from Lushan, Henan province. The genome is 152,642 bp in length and has a conserved structure that includes a pair of IR regions (25,726 bp), a LSC region (83,527 bp) and a SSC region (17,663 bp). The chloroplast genome contains 113 unique genes, four rRNA genes, 30 tRNA genes, and 79 protein-coding genes, 23 of which contain introns. The protein-coding genes account for a total of 24,412 codons, and most of them are A/T biased usage. We identified 32 simple sequence repeats (SSRs) and 48 long repeats. Furthermore, we developed valuable chloroplast molecular resources by comparing chloroplast genomes from three *Isodon* species, and both mVISTA and DnaSP analyses showed that *rps16-trnQ*, *trnS*-*trnG*, and *ndhC*-*trnM* are candidate regions that will allow the identification of intraspecific differences within *I*. *rubescens*. Also 14 candidate fragments can be used to identify interspecific differences between species in *Isodon*. A phylogenetic analysis of the complete chloroplast genomes of 24 species in subfamily Nepetoideae was performed using the maximum likelihood method, and shows that *I*. *rubescens* clustered closer to *I*. *serra* than *I*. *lophanthoides*. Interestingly, our analysis showed that *I*. *rubescens* (MW018469.1) from Xianyang, Shaanxi Province (*IR-X*), is closer to *I*. *serra* than to the other two *I*. *rubescens* accessions. These results strongly indicate that intraspecific diversity is present in *I*. *rubescens*. Therefore, our results provide further insight into the phylogenetic relationships and interspecific diversity of species in the genus *Isodon*.

## Introduction

*Isodon rubescens* (Hemsley) H. Hara is an important medical herbs classified in the tribe *Ocimeae* of the botanical family Lamiaceae [1]. *I*. *rubescens* possesses anti-cancer, anti-bacterial, anti-inflammatory, heat-clearing and detoxification activities. In addition, it activates blood circulation, and has traditionally been used to treat inflammation, respiratory and gastrointestinal bacterial infections, and malignant tumors [2]. The pharmacologically active ingredients known from *I*. *rubescens* consist of several diterpenoids such as oridonin and ponicidin, that possess a broad spectrum of clinical antitumor activities [3]. Furthermore, with its vigor and well-developed root system, *I*. *rubescens* is both drought-resistant and cold-resistant. Plantings of *I*. *rubescens* can be used to prevent erosion, conserve water and soil, and are also highly ornamental [4]. Because of its important medicinal value and wide geographical distribution in China, *I*. *rubescens* is an attractive study subject. Previous studies have shown that the contents of the main active components in *I*. *rubescens* from different geographical regions are significantly different, and its quality as a traditional Chinese medicine can thus be greatly affected. Phylogenetic studies using nuclear markers (microsatellites) have shown that *I*. *rubescens* populations display geographical diversification, and intraspecific variation [5, 6]. Therefore, a way to effectively classify the intraspecific resources of *I*. *rubescens* is the key to ensuring the medicinal quality of this herb.

In recent years, the rapid development of massively high-throughput DNA sequencing technologies and the implementation of many whole genome sequencing projects has led to a huge increase in the amount of available genomic data, and genome sequences are frequently used to determine phylogenetic relationships, patterns of evolution, and genetic diversity [7]. Chloroplast genomes have many advantages for these types of analyses, such as a small size, low nucleotide substitution rate, uniparental inheritance, and a highly conserved genomic structure when compared with mitochondrial and nuclear genomes; thus, chloroplast DNA (cpDNA) is widely used for species identification and evolutionary analysis [8, 9].

Chloroplasts are organelles in plant cells that contain chlorophyll, and they convert light energy from the sun into chemical energy via photosynthesis, which supplies the energy necessary for all of the biochemical processes required for plant growth and development [10, 11]. Chloroplasts have circular genomes with highly conserved gene order and content, and they range from 120 to 160 kb in size [12]. Chloroplast genomes encode 110–130 unique genes that include many genes for photosynthetic proteins, transfer RNA, ribosomal proteins, and chloroplastic RNA polymerase [13]. The typical angiosperm chloroplast genome has a conserved quadripartite structure that consists of two copies of an inverted repeat region (IRA and IRB) separated by one small single copy region (SSC) and one large single copy region (LSC). Chloroplast genomes are highly conserved, have a low rate of evolution, and do not undergo recombination; therefore, cpDNA can reflect kinship and the evolutionary relationships between related species. For these reasons, chloroplast DNA has been widely used to explore phylogenetic relationships in many diverse plant groups. For example, four medicinal *Salvia* species were compared by analysis of their chloroplast genomes [14], and an updated tribal classification for the Lamiaceae was constructed based on plastome phylogenomics [15]. Therefore, a comparative analysis of the chloroplast genome of *I*. *rubescens* can provide further insight into the phylogenetic relationships and interspecific diversity of species in the genus *Isodon*.

In this study, we sequenced the chloroplast genome of *I*. *rubescens* from Lushan, Henan province (*IR-L*) using Illumina DNA sequencing technology followed by reference-guided assembly of the *de novo* contigs. Furthermore, the chloroplast genome sequences of *Isodon* species including *I*. *rubescens* (MW376483.1) from Jiyuan, Henan Province (*IR-J*), *I*. *rubescens* (MW018469.1) from Xianyang, Shaanxi Province (*IR-X*), *I*. *lophanthoides* (MT317098.1), and *I*. *serra* (MT317099.1) were all taken from NCBI. We compared these five chloroplast genomes and show how they relate to other species within the genus *Isodon*. In addition, the complete cpDNA sequences from an additional 19 species in the subfamily Nepetoideae and one cpDNA sequence from *Scutellaria baicalensis*, another species in the Lamiales used in Chinese traditional medicine as an outgroup, were selected to construct a phylogenetic tree which was then used to explore the genetic relationships among species in subfamily Nepetoideae.

## Materials and methods

### Plant materials, DNA extraction, and DNA sequencing

Fresh leaves were collected from *I*. *rubescens* plants grown in the medical plants garden, Henan University of Traditional Chinese Medicine, and this germplasm was collected from Lushan, Henan province, China (*IR-L*). Voucher specimens were deposited at the specimen room of the School of Pharmacy, Henan University of Traditional Chinese Medicine. Total genomic DNA was extracted from the 100 mg fresh leaves using the Rapid Plant Genomic DNA Isolation Kit (Sangon, China) according to the manufacturer’s instructions. DNA concentration was 57.6 ng/μl, the OD_260/280_ was 1.93, and the DNA appeared as a discrete band when examined via agarose gel electrophoresis. Subsequently, the DNA samples were fragmented using a Covaris Ultrasonic Processor, and Hieff NGS^™^ DNA Selection Beads (Yeasen Biotechnology; catalog number 12601ES56) were used for fragment concentration and recovery. Illumina DNA sequencing libraries were than constructed after end repair, addition of dA to the 3’ ends of the fragments, ligating the sequencing adapters, and library amplification using the NEB Next^®^ Ultra^™^ DNA Library Prep Kit for Illumina^®^ (New England Bioldabs; E7370). The library fragment sizes were determined by agarose gel electrophoresis, the DNA fragment concentrations were quantified with a Qubit 4.0 fluorometer (Thermo Fisher), and the library DNA fragment size distribution was determined using the 2100 Bioanalyzer System with a DNA 1000 kit (Agilent Technologies). Finally, the qualified library was sequenced in 150-bp paired-end (PE) reads on the Illumina Hiseq 2500 platform.

### Genome assembly, annotation, and analysis

For quality control of the raw paired-end Illumina reads, FastQC was used to evaluate the quality of the raw data, and Trimmomatic was used to remove low quality reads and adapter sequences. Basic information such as read quality scores, sequence GC content, and adapter content were determined to assess the data quality. After obtaining high-quality sequencing data, referring to the published *I*. *serra* chloroplast genome (MT317099.1), we used the chloroplast DNA-like reads to assemble sequences with GetOrganelle v1.7.5 and NOVOPlasty to perform *de novo* assembly. NOVOPlasty, an program that assembles organellar genomes from whole-genome sequencing data, was able to assemble partial contigs and extend them until a circular genome became apparent [16]. PrInSeS-G (Version 1.0.0. beta) was used to correct base errors and insertional loss of small fragments that occurred during the assembly process [17]. Reverse BLAST searches were used to compare the assembled cpDNAs with reference genomes or built-in databases to optimize and extract assembly results. The chloroplast genomes were annotated using the Dual Organellar GenoMe Annotator (DOGMA) [18]. Putative start and stop codons together with intron positions were manually corrected based on comparisons with homologous genes from other sequenced chloroplast genomes. Hmmer v3.1b2 was used to predict rRNA genes, aragorn V1.2.38 was used to predict tRNAs, and all identified tRNAs were further verified by tRNAscan-SE [19]. The circular map of the *I*. *rubescens* chloroplast genome was drawn using the OGDRAW version 1.3.1 program [20].

### Repeat sequences, SSRs, and codon usage analysis

The online, web-based version of REPuter (https://bibiserv.cebitec.uni-bielefeld.de/reputer/) was used to detect sequence repeats in the cpDNA assemblies including forward, palindromic, reverse, and complementary repeats. The minimal repeat size was set to 18 bp, and the sequence identity was > 90% [21]. Simple sequence repeats (SSRs) were identified by MISA-web (https://webblast.ipk-gatersleben.de/misa/) with the minimum number of mono-, di-, tri-, tetra-, penta- and hexanucleotide repeats set to 10, 6, 5, 5, 5, and 5, respectively [22]. To avoid sampling errors, protein-coding genes were selected for synonymous codon usage analysis using the program CodonW1.4.2 (http://downloads.fyxm.net/CodonW-76666.html). The overall codon usage and the relative synonymous codon usage (RSCU) were analyzed.

### Comparative analysis of chloroplast genomes

The complete chloroplast genome of *I*. *rubescens* (*IR-L*) was compared with the chloroplast genomes of *I*. *rubescens* (MW376483.1) from Jiyuan, Henan Province (*IR-J*), *I*. *rubescens* (MW018469.1) from Xianyang, Shaanxi Province (*IR-X*), *I*. *serra* (MT317099.1), and *I*. *lophanthoides* (MT317098.1) in the *Isodon* genus using mVISTA (http://genome.lbl.gov/vista/index.shtml) with the LAGAN mode [23]. The annotation of *I*. *rubescens* cpDNA was used as the reference. The comparison of the LSC/IRB/SSC/IRA boundaries among the five *Isodon* chloroplast genome sequences was performed with IRSCOPE (https://irscope.shinyapps.io/irapp/) based on the annotations of the chloroplast genomes available in GenBank. The single nucleotide polymorphisms (SNPs) and insertion/deletions (indels) were recorded separately as well as their locations by BWA SAMtools in the chloroplast genome [24].

### Phylogenetic analysis

To determine the phylogenetic position of *I*. *rubescens*, the complete chloroplast genome sequences of 24 species in the subfamily Nepetoideae (Lamiaceae) and the *Scutellaria baicalensis* chloroplast genome as the outgroup were downloaded from GenBank. Sequences were aligned using the MAFFT algorithm on the MAFFT version 7 alignment server [25]. A maximum likelihood (ML) phylogenetic tree was generated with 1,000 bootstrap replicates using MEGA 6 software [26].

## Results and discussion

### Features of the *I*. *rubescens* chloroplast genome

**Fig 1:**
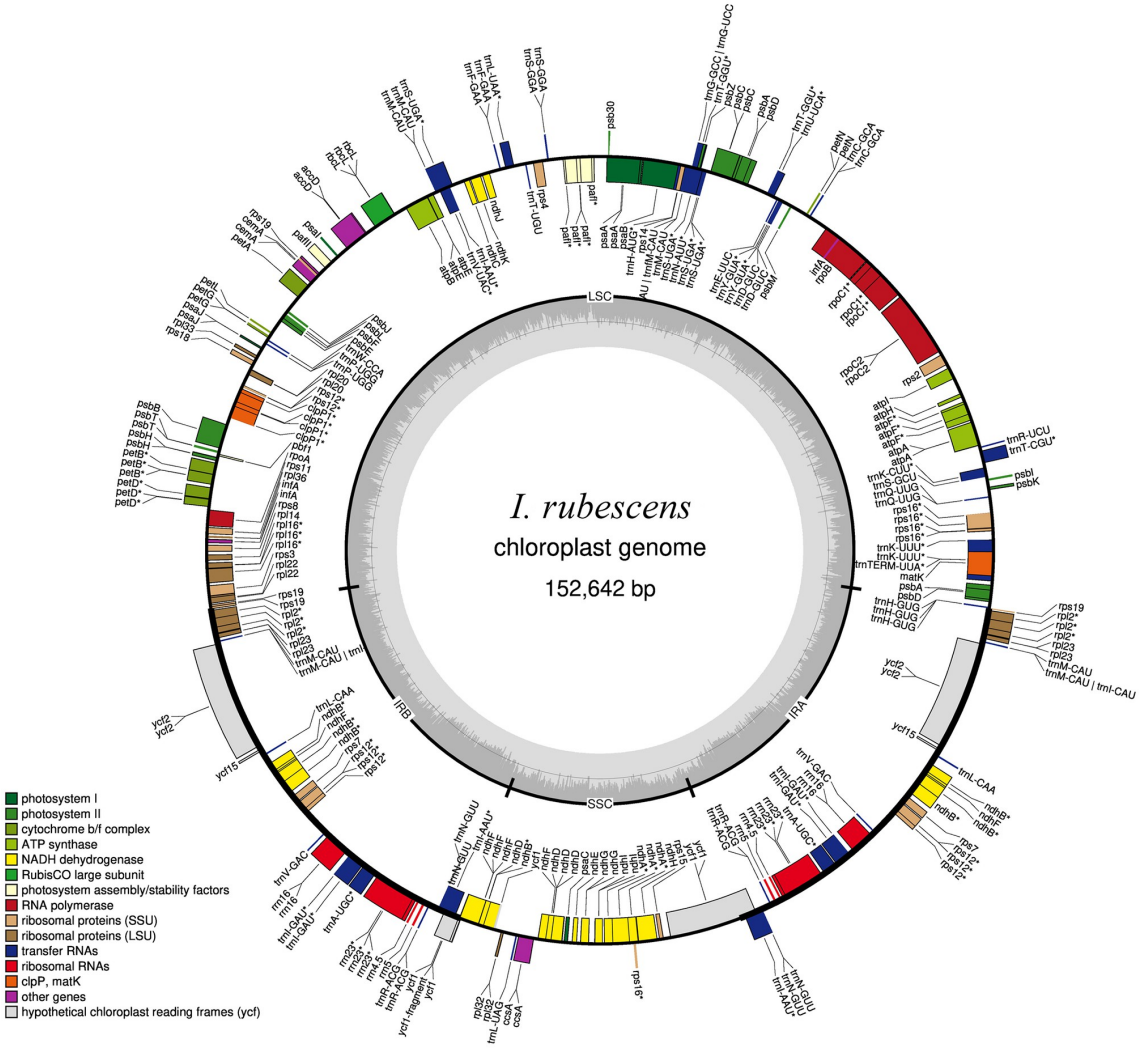
Circular gene map of the *I*. *rubescens* chloroplast genome. Genes inside the circle are transcribed clockwise, and those outside are transcribed counter clockwise. Groups of genes with different functions are color-coded. The darker gray in the inner circle shows the GC content, while the lighter gray shows the AT content.

Raw data consisting of 26.93 million total reads and 3.9 Gb total bases (90.06% with quality scores ≥Q30) was obtained from *I*. *rubescens* by paired-end sequencing (PE150) on an Illumina HiSeq platform (S1 Table). All of the read data was deposited in the NCBI Sequence Read Archive (SRA) under accession number MW940491.1. The size of the complete chloroplast genome assembly was 152,642 bp (Fig 1). The *I*. *rubescens* chloroplast genome has a typical quadripartite structure, including a pair of IRs (25,726 bp) separated by the LSC (83,527 bp) and SSC (17,663 bp) regions (Fig 1). Previous studies have shown that the GC content is predicted to significantly affect genome function and species ecology, and it can be an important indicator of species affinity [27]. Therefore, we analyzed the GC contents of the LSC, SSC, IR regions, and the whole genome and found them to be 35.6%, 31.0%, 43.1%, and 37.6%, respectively (S2 Table), which is similar to the GC contents reported for the chloroplast genomes of other *Isodon* species [28]. Our results also revealed that the GC contents of the IR region are higher than those of the LSC and SSC regions, which is similar to findings in other plants [29].

Plant chloroplast genomes contain between 110 and 130 unique genes, such as genes that encode photosynthetic proteins, self-replication expression-related genes, and some other protein-coding genes [30]. In the *I*. *rubescens* chloroplast genome, 113 unique genes out of a total of 133 predicted functional genes included four rRNA genes, 30 tRNA genes, and 79 protein-coding genes that were divided into four groups; photosynthetic genes (45 genes), self-replication expression-related genes (59 genes), other genes (five genes), and genes of unknown function (four genes) (Table 1). Of these, 18 genes are repeated in the IR regions (seven protein-coding, seven tRNA, and four rRNA genes). A total of 83 genes, 61 protein-coding and 22 tRNA genes, are present in the LSC region while 11 protein-coding genes and one tRNA gene are present in the SSC region. Specifically, one *ycf1* pseudogene and one *rps19* pseudogene are located in the two IR boundary regions (Fig 1). This phenomenon is very similar to findings in other plants species [31].

**Table 1:** List of genes in the chloroplast genome of *I*. *rubescens*.

Category for genes	Group of genes	Name of genes
**Photosynthesis**	Subunits of photosystem I	*psaA*, *psaB*, *psaC*, *psaI*, *psaJ*, *ycf3*^b^, *ycf4*
	Subunits of photosystem II	*psbA*, *psbB*, *psbC*, *psbD*, *psbE*, *psbF*, *psbH*, *psbI*, *psbJ*, *psbK*, *psbL*, *psbM*, *psbT*, *psbZ*
	Subunits of cytochrome b/f complex	*petA*, *petB*^b^, *petD*^b^, *petG*, *petL*, *petN*
	Large subunit of Rubisco	*rbcL*
	Subunits of ATP synthase	*atpA*, *atpB*, *atpE*, *atpF1*, *atpH*, *atpI*
	Subunits of NADH-dehydrogenase	*ndhA*^b^, *ndhB*^a^^,^^b^, *ndhC*, *ndhD*, *ndhE*, *ndhF*, *ndhG*, *ndhH*, *ndhI*, *ndhJ*, *ndhK*
**Self-replication**	Ribosomal RNA genes	*rrn16*^a^, *rrn23*^a^, *rrn5*^a^, *rrn4*.*5*^a^
	Transfer RNA genes	*trnA-UGC*^a^^,^^b^, *trnC-GCA*, *trnD-GUC*, *trnE-UUC*, *trnF-GAA*, *trnG-GCC*, *trnG-UCCb*, *trnH-GUG*, *trnI-CAU*^a^, *trnI-GAU*^a^^,^^b^, *trnK-UUUb*, *trnL-CAA*^a^, *trnL-UAAb*, *trnL-UAG*, *trnM-CAU*, *trnfM-CAU*, *trnN-GUU*^a^, *trnP-UGG*, *trnQ-UUG*, *trnR-ACG*^a^, *trnR-UCU*, *trnS-GCU*, *trnS-GGA*, *trnS-UGA*, *trnT-GGU*, *trnT-UGU*, *trnV-GAC*^a^, *trnV-UAC*^b^, *trnW-CCA*, *trnY-GUA*
	Small subunit of ribosome	*rps2*, *rps3*, *rps4*, *rps7*^a^, *rps8*, *rps11*, *rps12*^a^^,^ ^b^^,^ ^c^, *rps14*, *rps15*, *rps16*^b^, *rps18*, *rps19*^a^
	Large subunit of ribosome	*rpl2*^a^^,^ ^b^, *rpl14*, *rpl16*^b^, *rpl20*, *rpl22*, *rpl23*^a^, *rpl32*, *rpl33*, *rpl36*
	DNA-dependent RNA polymerase	*rpoA*, *rpoB*, *rpoC1*^b^, *rpoC2*
**Other genes**	Maturase	*matK*
	Envelope membrane protein	*cemA*
	Subunit of acetyl-CoA	*accD*
	C-type cytochrome synthesis gene	*ccsA*
	Protease	*clpPb*
**unknown function**	Conserved Open reading frames	*ycf1*^a^, *ycf2*^a^, *infA*, *pbf1*

^a^. Two gene copies.

^b^. Genes containing introns.

^c^. Genes divided into two independent transcription units.

Introns play a crucial role in “intron-mediated enhancement”, which increases gene expression and enhances the efficiency of mRNA translation [32]. Introns are also present in some chloroplast genes and play an important role in the regulation of gene expression [33]. A total of 23 genes containing introns were identified in the *I*. *rubescens* chloroplast genome. Nineteen of these genes contain one intron and four of them contain two introns (S3 Table). The intron in the *trnK*-*UUU* gene is the longest having 2,504 bp, and contains the *matK* gene. The 5’ end of the *rps12* gene is located in the LSC region, while the 3’ end is located in the IR regions, making it a trans-spliced gene. This is a general phenomenon of intron distribution in chloroplast genomes [14]. In addition, intron lengths range from is 459–1,032 bp, except for the longest intron (2,504 bp), and the average intron length was 797 bp in the chloroplast genome of *I*. *rubescens* (S3 Table).

### Codon usage

The genetic code plays an important role in the transmission of genetic information and is the link between the nucleic acids and the proteins they encode in all organisms [34]. Different genomes show synonymous codon usage bias (SCUB) in which synonymous codons are not used equally in translation [35]. In the chloroplast genome, codon usage plays an important role in evolution [36]. Thus, we measured the codon usage frequency and relative synonymous codon usage (RSCU) frequency for all the protein-coding genes in the *I*. *rubescens* chloroplast genome (Table 2). A total of 73,242 bp in all the protein-coding genes accounted for 24,412 codons. Of these codons, 2,571 (10.53%) encoded leucine, whereas only 268 (1.10%) encoded cysteine, representing the most and the least frequently used amino acids in proteins encoded in the *I*. *rubescens* chloroplast genome, similar to what is found in other chloroplast genomes [37]. When comparing the 30 tRNAs genes in the *I*. *rubescens* chloroplast genome, the codon-anticodon recognition patterns showed that these 30 tRNAs correspond to all 20 amino acids. Previous studies have shown that the codon composition of genes in the chloroplast genome has an A/T bias, especially at the third codon position, which is widely found in many land plants [38]. In our study, the A/T contents at the first, second, and third codon positions were 54.28%, 61.65%, and 70.76%, respectively. In addition, the RSCU values of 30 codons were >1, indicating that these codons show biased usage. Except for the leucine TTG codon that ends in G, all the biased usage codons had A/T at the third position. The use of ATG for methionine and TGG for tryptophan showed no biased usage (RSCU = 1) (Table 2).

**Table 2:** Codon-anticodon recognition patterns and codon usage in the chloroplast genome of *I*. *rubescens*.

Amino Acid	Codon	No.	RSCU	tRNA	Amino Acid	Codon	No.	RSCU	tRNA
Ala	GCU	598	1.77		Arg	CGU	325	1.32	trnR-ACG
Ala	GCC	219	0.65		Arg	CGC	112	0.46	
Ala	GCA	382	1.13	trnA-UGC	Arg	CGA	330	1.34	
Ala	GCG	150	0.44		Arg	CGG	118	0.48	
Ile	AUU	1055	1.5		Arg	AGA	454	1.85	trnR-UCU
Ile	AUC	424	0.6	trnI-GAU	Arg	AGG	137	0.56	
Ile	AUA	627	0.89		Asn	AAU	894	1.54	
Leu	UUA	844	1.97	trnL-UAA	Asn	AAC	265	0.46	trnN-GUU
Leu	UUG	502	1.17	trnL-CAA	Asp	GAU	754	1.62	
Leu	CUU	537	1.25		Asp	GAC	179	0.38	trnD-GUC
Leu	CUC	160	0.37		Cys	UGU	207	1.54	
Leu	CUA	369	0.86	trnL-UAG	Cys	UGC	61	0.46	trnC-GCA
Leu	CUG	159	0.37		Gln	CAA	653	1.53	trnQ-UUG
Met	AUG	586	1	trnI-CAU, trnM-CAU, trnfM-CAU	Gln	CAG	199	0.47	
Pro	CCU	388	1.51		Glu	GAA	977	1.56	trnE-UUC
Pro	CCC	212	0.82		Glu	GAG	278	0.44	
Pro	CCA	284	1.1	trnP-UGG	Gly	GGU	528	1.25	
Pro	CCG	145	0.56		Gly	GGC	188	0.44	trnG-GCC
Ser	UCU	537	1.73		Gly	GGA	690	1.63	trnG-UCC
Ser	UCC	294	0.95	trnS-GGA	Gly	GGG	290	0.68	
Ser	UCA	364	1.18	trnS-UGA	His	CAU	437	1.53	
Ser	UCG	166	0.54		His	CAC	134	0.47	trnH-GUG
Ser	AGU	390	1.26		Lys	AAA	1005	1.51	trnK-UUU
Ser	AGC	107	0.35	trnS-GCU	Lys	AAG	326	0.49	
Thr	ACU	503	1.61		Phe	UUU	945	1.38	
Thr	ACC	243	0.78	trnT-GGU	Phe	UUC	428	0.62	trnF-GAA
Thr	ACA	375	1.2	trnT-UGU	TER	UAA	46	1.6	
Thr	ACG	129	0.41		TER	UAG	24	0.84	
Val	GUU	504	1.48		TER	UGA	16	0.56	
Val	GUC	157	0.46	trnV-GAC	Trp	UGG	421	1	trnW-CCA
Val	GUA	543	1.59	trnV-UAC	Tyr	UAU	714	1.63	
Val	GUG	161	0.47		Tyr	UAC	163	0.37	trnY-GUA

### SSRs and long-repeat analysis

Simple sequence repeats (SSRs), also known as microsatellites, have a relatively high rate of mutation and copy number polymorphisms, making them valuable molecular markers for genetic diversity studies, polymorphism investigations, evolutionary studies, and plant breeding. Compound SSRs also can provide insight into the evolution of microsatellites [39, 40]. In the *I*. *rubescens* chloroplast genome, a total of 32 SSRs, including 29 mononucleotides, one dinucleotide, and two compound SSRs with a length of at least 10 bp, were identified, and no trinucleotide repeats were found (Table 3). In our results, all mononucleotides SSRs were either poly A or poly T, and all dinucleotides were made up of AT/TA. This result agrees with a previous finding that the most abundant SSRs consist of short polyadenine (polyA) or polythymine (polyT) repeats in chloroplast genomes [41], and the two compound SSRs had lengths of 54 bp and 23 bp. Most of the identified SSRs were within noncoding DNA regions; 25 were located in the intergenic regions, four were located in introns, and three were located in the *rpoC2*, *atpB*, and *ycf1* genes (Table 3).

**Table 3:** SSRs in the chloroplast genome of *I*. *rubescens*.

SSR nr.	SSR type	SSR	size	start	end	Region	Position
**1**	p1	(A)11	11	4444	4454	LSC	IGS
**2**	p1	(A)10	10	6668	6677	LSC	IGS
**3**	p1	(A)10	10	8147	8156	LSC	IGS
**4**	c	(T)12gaaaagaaaaaaaataatgccttttttttaag(T)10	54	11734	11787	LSC	IGS
**5**	c	(T)10c(A)12	23	16305	16327	LSC	IGS
**6**	p1	(T)11	11	18536	18546	LSC	rpoC2
**7**	p1	(T)11	11	28760	28770	LSC	IGS
**8**	p1	(T)12	12	35002	35013	LSC	IGS
**9**	p1	(A)14	14	36248	36261	LSC	trnH-AUG(intron)
**10**	p1	(T)12	12	41884	41895	LSC	IGS
**11**	p1	(A)10	10	44220	44229	LSC	IGS
**12**	p1	(T)13	13	46005	46017	LSC	IGS
**13**	p2	(TA)7	14	46164	46177	LSC	IGS
**14**	p1	(T)10	10	46693	46702	LSC	IGS
**15**	p1	(A)11	11	46869	46879	LSC	IGS
**16**	p1	(T)10	10	54295	54304	LSC	atpB
**17**	p1	(T)10	10	58922	58931	LSC	IGS
**18**	p1	(T)13	13	59595	59607	LSC	IGS
**19**	p1	(T)10	10	60808	60817	LSC	IGS
**20**	p1	(T)10	10	63527	63536	LSC	IGS
**21**	p1	(T)11	11	67056	67066	LSC	IGS
**22**	p1	(T)13	13	69185	69197	LSC	IGS
**23**	p1	(T)10	10	70138	70147	LSC	clpP1(intron)
**24**	p1	(A)11	11	70426	70436	LSC	clpP1(intron)
**25**	p1	(T)11	11	71303	71313	LSC	clpP1(intron)
**26**	p1	(A)10	10	73740	73749	LSC	IGS
**27**	p1	(T)12	12	79583	79594	LSC	IGS
**28**	p1	(T)10	10	98083	98092	IRB	IGS
**29**	p1	(T)10	10	112297	112306	SSC	IGS
**30**	p1	(T)10	10	117749	117758	SSC	IGS
**31**	p1	(T)11	11	125193	125203	SSC	ycf1
**32**	p1	(A)10	10	138158	138167	IRA	IGS

Repeat sequences are widely distributed in the genome, and play an important part in genome rearrangements [42]. A total of 48 repeats were found in the *I*. *rubescens* chloroplast genome, including 15 forward, 20 palindromic, 11 reverse repeats and two complementary repeats (Table 4). Of the 48 repeats, 32 were 20–29 bp in length and 16 were 18–19 bp in length. In addition, 26 of the repeats were concentrated in the LSC region, six repeats were located in the IRs, and three were located in IRA, IRB, and the SSC, respectively. These SSRs and the repeats identified in the *I*. *rubescens* chloroplast genome can be used as a significant molecular markers to explore the genetic diversity and species differentiation in *I*. *rubescens* and related species in future research.

**Table 4:** Long repeat sequences identified in the chloroplast genome of *I*. *rubescens*.

No.	Repeat size (bp)	Type	Repeat 1 start	Repeat 2 start	Mismatch(bp)	E-Value	Region
**1**	29	P	8217	45035	0	2.27×10^−08^	LSC
**2**	26	P	87192	87192	0	1.46×10^−06^	IRB
**3**	26	F	87192	149031	0	1.46×10^−06^	IRB;IRA
**4**	26	F	125658	125676	0	1.46×10^−06^	SSC
**5**	26	P	149031	149031	0	1.46×10^−06^	IRA
**6**	24	P	74746	74746	0	2.33×10^−05^	LSC
**7**	24	P	114085	114132	0	2.33×10^−05^	SSC
**8**	23	P	29238	29263	0	9.31×10^−05^	LSC
**9**	22	F	9802	36047	0	3.72×10^−04^	LSC
**10**	22	P	30490	30490	0	3.72×10^−04^	LSC
**11**	22	F	45998	59588	0	3.72×10^−04^	LSC
**12**	22	F	46185	46215	0	3.72×10^−04^	LSC
**13**	22	P	46808	46808	0	3.72×10^−04^	LSC
**14**	22	F	90824	90842	0	3.72×10^−04^	IRB
**15**	22	P	90824	145385	0	3.72×10^−04^	IRB, IRA
**16**	22	P	90842	145403	0	3.72×10^−04^	IRB, IRA
**17**	22	F	97710	118963	0	3.72×10^−04^	IRB, SSC
**18**	22	P	118963	138517	0	3.72×10^−04^	IRA, SSC
**19**	22	F	145385	145403	0	3.72×10^−04^	IRA
**20**	21	F	8222	35143	0	1.49×10^−03^	LSC
**21**	21	C	18527	46860	0	1.49×10^−03^	LSC
**22**	21	R	31283	31283	0	1.49×10^−03^	LSC
**23**	21	P	35143	45038	0	1.49×10^−03^	LSC
**24**	21	F	36272	66485	0	1.49×10^−03^	LSC
**25**	20	P	8649	8649	0	5.96×10^−03^	LSC
**26**	20	R	8853	8853	0	5.96×10^−03^	LSC
**27**	20	R	11769	117746	0	5.96×10^−03^	LSC
**28**	20	F	38272	40496	0	5.96×10^−03^	LSC
**29**	20	R	50182	50182	0	5.96×10^−03^	LSC
**30**	20	P	51977	102498	0	5.96×10^−03^	LSC, IRB
**31**	20	F	51977	133731	0	5.96×10^−03^	IRB, IRA
**32**	20	P	73915	73939	0	5.96×10^−03^	LSC
**33**	19	R	1790	14187	0	2.38×10^−02^	LSC
**34**	19	C	7309	47088	0	2.38×10^−02^	LSC
**35**	19	R	15030	15030	0	2.38×10^−02^	LSC
**36**	19	P	30326	115836	0	2.38×10^−02^	LSC, SSC
**37**	19	R	46864	46864	0	2.38×10^−02^	LSC
**38**	19	P	73723	122022	0	2.38×10^−02^	LSC, SSC
**39**	19	F	90786	90804	0	2.38×10^−02^	IRB
**40**	19	P	90786	145426	0	2.38×10^−02^	IRB, IRA
**41**	19	P	90804	145444	0	2.38×10^−02^	IRB, IRA
**42**	19	R	118508	118508	0	2.38×10^−02^	SSC
**43**	19	F	145426	145444	0	2.38×10^−02^	IRA
**44**	18	R	148	69617	0	9.54×10^−02^	LSC
**45**	18	R	4515	112976	0	9.54×10^−02^	LSC, SSC
**46**	18	F	6222	27249	0	9.54×10^−02^	LSC
**47**	18	R	6251	6251	0	9.54×10^−02^	LSC
**48**	18	P	7904	112977	0	9.54×10^−02^	LSC, SSC

F: forward repeats, P: palindromic repeats, R: reverse repeats, C: complementary repeats

### Inter- and intra-specific comparative analysis of genomic structure

Chloroplast DAN sequences are often used in phylogenetic studies and to measure the genetic diversity within a species [43]. Previous studies have shown that *I*. *rubescens* has intraspecific variation [5]. Thus, it is necessary to understand the interspecific and intraspecific differences between the chloroplast genomes in *I*. *rubescens*. The sequences of chloroplast genomes from two other collections of *I*. *rubescens*, *IR-X* (MW018469.1) and *IR-J* (MW376483.1), *I*. *lophanthoides* (MT317098.1), and *I*. *serra* (MT317099.1) were obtained from NCBI. The results of comparisons showed that *I*. *lophanthoides* has the smallest chloroplast genome with the largest SSC region (17,699 bp) and the smallest LSC (83,095 bp) and IR regions (17,699 bp), and *IR-X* has the largest chloroplast genome with the largest LSC region (83,656 bp) (S4 Table). These results indicate that there are significant interspecific sequence differences in the cpDNA of *Isodon* species. In addition, comparative analysis of the three *I*. *rubescens* cp genome sequences showed that the number of genes and the genome sizes including the lengths of the LSC, SSC, and IR regions are all different. These results indicate that there is also intraspecific variation in the cpDNA sequence *I*. *rubescens*.

**Fig 2:**
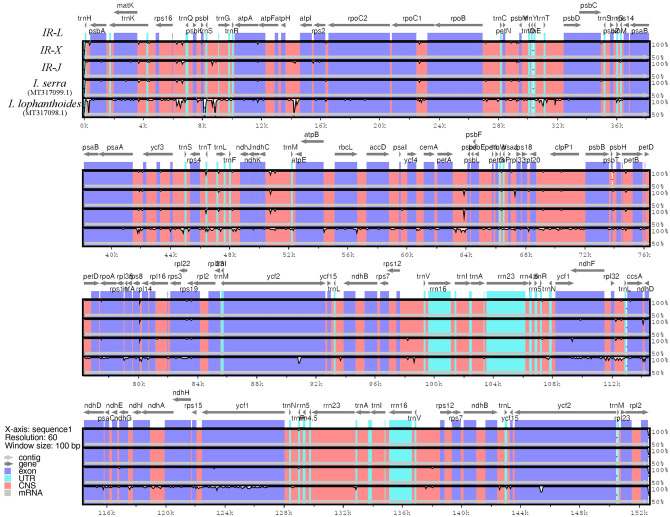
Sequence alignment of five *Isodon* chloroplast genomes using mVISTA. The annotation of *I*. *rubescens* (*IR-L*) was used as the reference. Gray arrows above the alignment indicate the position and direction of transcription of each gene. The scale on the vertical axes represents the percent sequence identity between 50% and 100%. *IR-J*, *IR-X*, and *IR-L* are the three accessions of *I*. *rubescens*.

To further analyze the potential divergence of these genome sequences, sequence identity was calculated by using the program mVISTA with *I*. *rubescens IR-L* as the reference (Fig 2). The results of this comparison revealed that the LSC regions are more divergent than are the SSC and IR regions, and that higher levels of sequence divergence are found in noncoding compared to coding regions. In the comparative analysis of three *I*. *rubescens* chloroplast genomes, relatively higher levels of variation were found in the *rps16*-*trnQ*, *trnS*-*trnG*, and *ndhC*-*trnM* regions (S1 Fig). Comparison of five chloroplast genome sequences from species of *Isodon* showed that *I*. *lophanthoides* is significantly different from the other fours, while the differences between the cpDNA sequences of *I*. *rubescens (IR-L)* and *I*. *serra* were small. Of the five chloroplast genomes, higher levels of variation were found in the coding regions of some genes, including *rpoC1*, *clpP1*, ccsA, *ndhF*, and *ycf1*. The highest divergence in noncoding regions was found in the intergenic regions *rps16*-*trnQ*, *trnS*-*trnG*, *atpH*-*atpI*, *trnE*-*trnT*, *psaA-ycf3*, *ndhC*-*trnM*, *petA*-*psbL*, *rlp20*-*clpP1*, and *rpl32-trnL*. These results are mostly consistent with those reported for the chloroplast genomes of other plant species [44].

**Fig 3:**
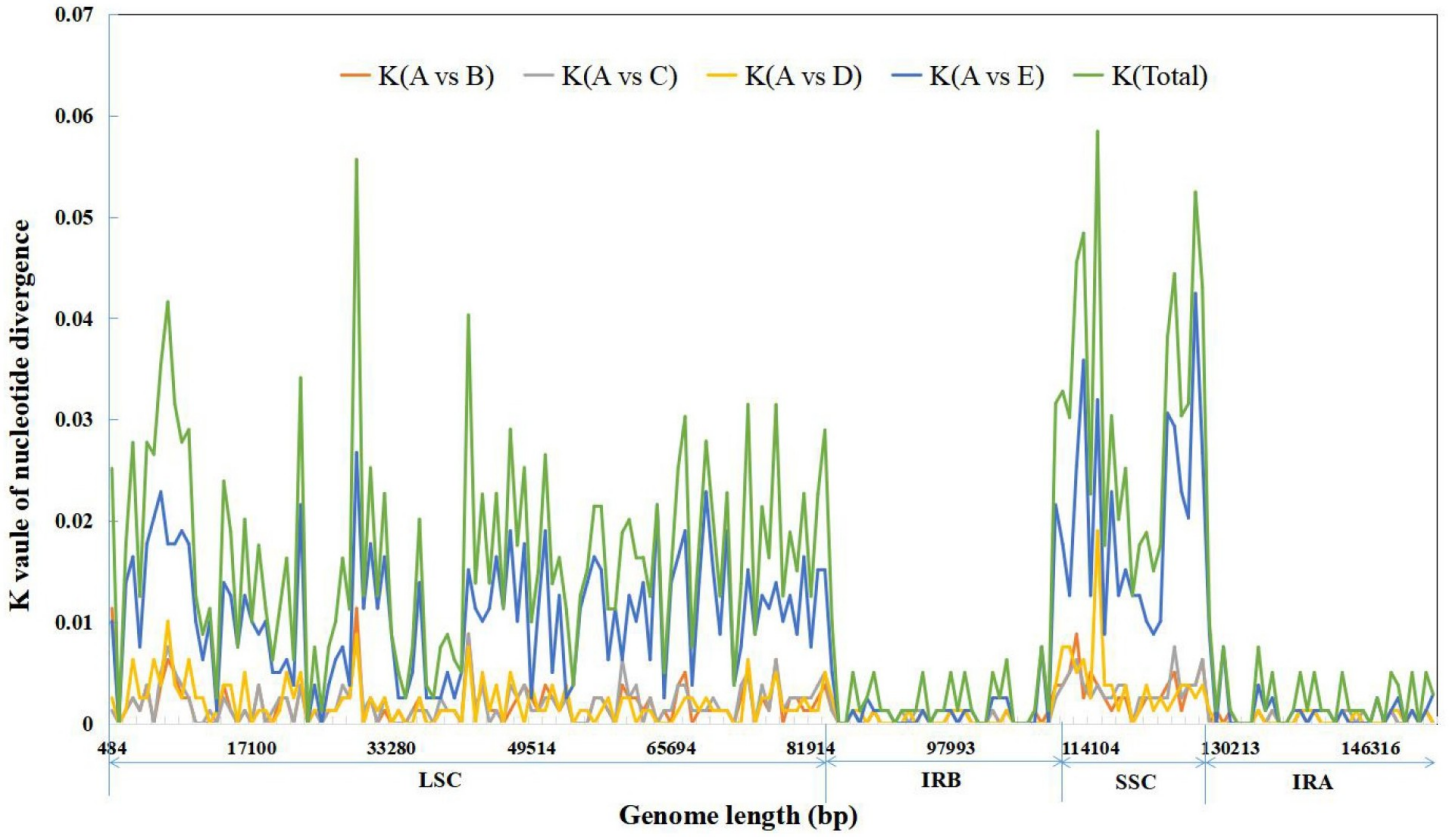
Sliding window analysis of five complete *Isodon* chloroplast genomes. Window length: 800 bp; step size: 200 bp. X-axis: nucleotide positions in the chloroplast genomes. Y-axis: K values of nucleotide diversity. A: *I*. *rubescens* from Lushan, Henan province (*IR-L*), B: *I*. *rubescens* (MW018469.1) from Xianyang, Shaanxi Province (*IR-X*), C: *I*. *rubescens* (MW376483.1) from Jiyuan, Henan Province (*IR-J*), D: *I*. *serra* (MT317099.1), E: *I*. *lophanthoides* (MT317098.1). Total: the total nucleotide diversity values for the A vs. B, A vs. C, A vs. D, and A vs. E comparisons.

Furthermore, the results of the sliding window analysis using DnaSP6 indicated that the nucleotide variation in the *Isodon* chloroplast genomes is mainly present in the LSC and SSC regions (Fig 3). There were nine mutational hotspots detected that showed remarkably higher K values (total values >0.04), including three regions (*petN*-*psbM*, *rps16*-*trnQ*, and *psaA*-*ycf3*) in the LSC and six regions (*ndhF*, *ccsA*, *rpl32-trnL*, and three regions in *ycf1*) in the SSC of the chloroplast genomes. These regions may be undergoing more rapid nucleotide substitution at the species level, indicating the potential application of molecular markers for phylogenetic analyses and plant identification in the genus *Isodon*. In addition, the average nucleotide diversity (K value) of *IR-L* compared with *IR-X*, *IR-J*, *I*. *serra* (MT317099.1), and *I*. *lophanthoides* (MT317098.1) was 0.00155, 0.00161, 0.00174, and 0.00856, respectively, and the total average value of nucleotide diversity was 0.00337. These results show that the intra-specific cpDNA sequence diversity in *I*. *rubescens* was the lowest, the interspecific cpDNA diversity between *I*. *rubescens* and *I*. *serra* was also small, but the interspecific nucleotide diversity between the chloroplast genomes of *I*. *rubescens* and *I*. *lophanthoides* was relatively large.

**Fig 4:**
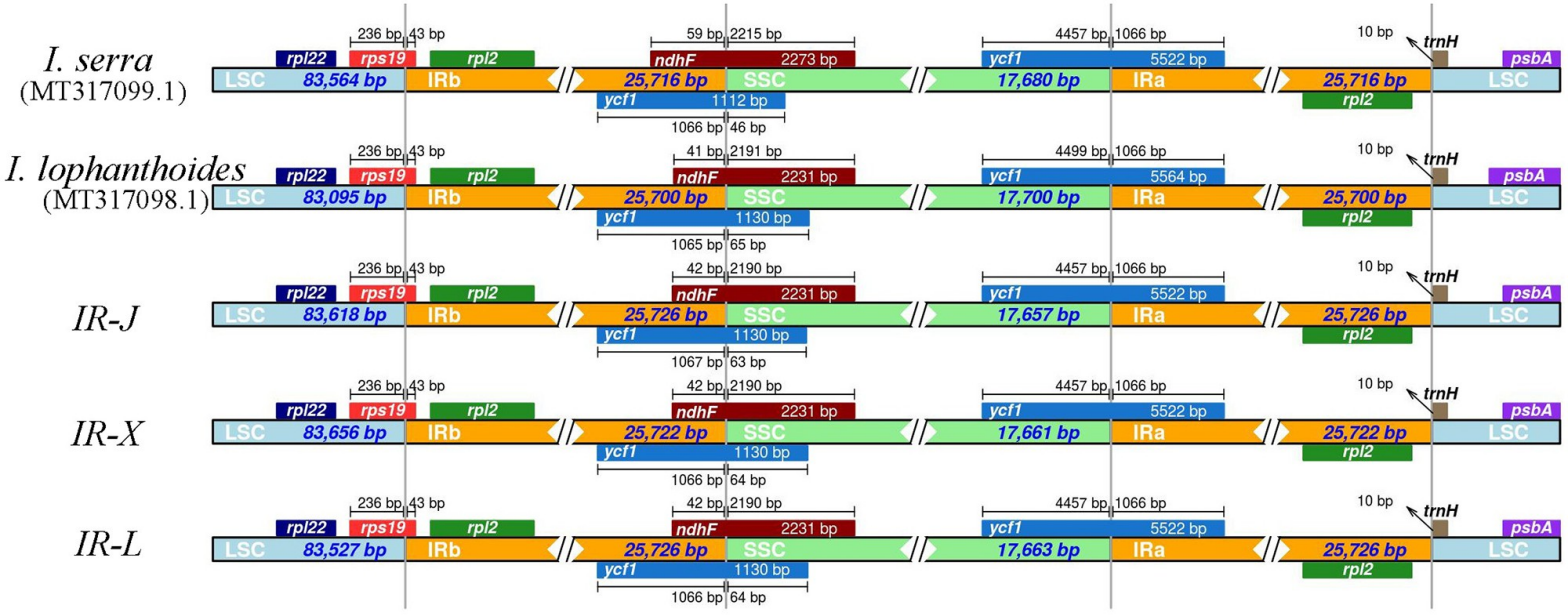
Comparison of the LSC, SSC, and IR border regions among the five *Isodon* chloroplast genomes of examined in this study. *IR-J*, *IR-X*, and *IR-L* are the three accessions of *I*. *rubescens*.

Previous studies have shown that there are variable patterns in the expansion and contraction of the IR region in the chloroplast genomes which can lead to size variation and rearrangement at the LSC/IRB/SSC/IRA boundaries [45]. In this study, detailed comparisons of the genes adjacent to the LSC/IRB/SSC/IRA boundaries were compared among the five *Isodon* chloroplast genomes (Fig 4). In the LSC/IRB boundary, there is a fragment of the *rps19* pseudogene, and there were no differences between the five genomes. In the IRB/SSC boundary, overlaps between the *ycf1* pseudogene and *ndhF* gene were detected, but the only differences were found in *I*. *serra*. In the chloroplast genome of *I*. *serra*, the *ndhF* gene terminates prematurely, which reduces its length by 28 bp, while the *ycf1* gene increased in length by 42 bp. In the SSC/IRA boundary, the *ycf1* pseudogene is present in all five cp genomes, and the only difference was that the *ycf1* gene increased in length by 42 bp in the SSC region of the *I*. *lophanthoides* cp genome. There were no differences detected in the IRA/SSC boundary regions. In conclusion, there were no differences detected in the boundariey regions in the three *I*. *rubescens* chloroplast genomes, but there were differences in the IRB/SSC and IRA/SSC boundaries between the *I*. *serra* and *I*. *lophanthoides* cp genomes. These results also show that there was expansion and contraction in the cpDNA boundary regions among the different *Isodon* species examined.

In addition, to detect the differences between *I*. *rubescens* and the two other *Isodon* species *I*. *serra* and *I*. *lophanthoides* at the cpDNA level, SNP and Indel analyses were also performed with the cpDNA sequence of *IR-L* as the reference. There were 102, 133, 201, and 654 SNPs in the cpDNA identified when *IR-L* was compared to the *IR-X*, IR-J, *I*. *serra*, and *I*. *lophanthoides* cp genomes, respectively. And there were 30, 62, 149, and 493 indels in the cpDNA identified when compared to *IR-X*, IR-J, *I*. *serra*, and *I*. *lophanthoides*, respectively (S5 Table). These SNPs and Indels will be very useful resources for phylogenetic analysis and species identification in the genus *Isodon*.

### Phylogenetic analysis

**Fig 5:**
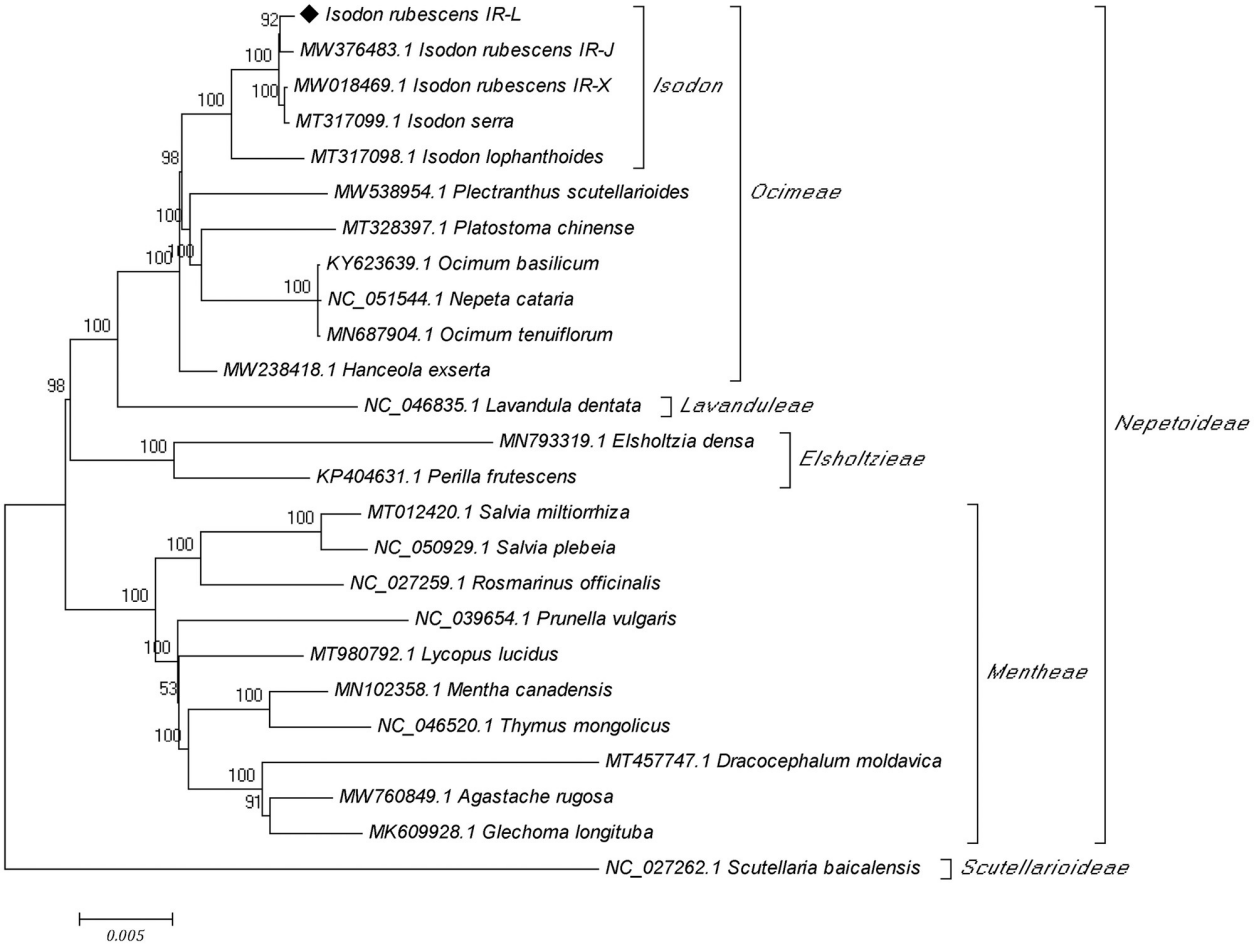
Phylogenetic tree based on the complete chloroplast genomes from 25 species constructed using the maximum likelihood method. The *Scutellaria baicalensis* cpDNA sequence was used as the outgroup. Bootstrap values are shown at the nodes.

Chloroplast genomes are widely used for phylogeny reconstruction and population analyses because of their ease of use and the abundant phylogenetic information they contain [37]. To better determine the phylogenetic position of *I*. *rubescens* and further clarify the evolutionary relationships within the subfamily Nepetoideae, we performed a phylogenetic analyses based on the complete chloroplast genome sequences of 24 Nepetoideae species using the chloroplast genome of *Scutellaria baicalensis* (subfamily Scutellarioideae) as the outgroup. In the resulting phylogenetic tree bootstrap (BS) analysis showed that 18 nodes were strongly supported with BS values ≥95%, and 16 nodes had BS values of 100% (Fig 5). The 24 species of Nepetoideae formed two major clades; the 10 species in tribe *Mentheae* comprised the smaller clade, and the 14 species/accessions from tribes *Ocimeae*, *Lavanduleae*, and *Elsholtzieae* grouped together in the larger clade. This result shows that species in tribes *Ocimeae*, *Lavanduleae*, and *Elsholtzieae* are more closely related evolutionarily to one another than they are to the species in tribe *Mentheae*. In the *Ocimeae*, there are two subclades, the smallest contains the single species in the genus *Hanceola*, and the largest contains all species in the genera *Isodon*, *Plectranthus*, *Platostoma*, and *Ocimum*. Furthermore, the three species in the genus *Isodon* define a well-supported clade that is separate from species in *Plectranthus*, *Platostoma*, and *Ocimum*. In addition, the *Isodon* clade, contains three accessions of *I*. *rubescens* and one each of *I*. *serra*, and *I*. *lophanthoides*. It is clear from this phylogenetic analysis that *I*. *lophanthoides* is evolutionarily distant from both *I rubescens* and *I*. *serra* and that *I*. *rubescens* is more closely related to *I*. *serra* than to *I*. *lophanthoides*. It is interesting that in the phylogenetic analysis, the three *I*. *rubescens* accessions are divided into two small branches, and one accession, *IR-X* (MW018469.1), shows a closer affinity to *I*. *serra* than it does to *IR-J* and *IR-L*. This result indicates that there could be considerable intraspecific variation present in *I*. *rubescens*, at least as determined by cpDNA analysis. Our results also show that the phylogenetic analysis of chloroplast genomes also can provide a reliable method for intraspecific identification and classification of geographical collections of *I*. *rubescens*.

## Conclusions

In this study, the chloroplast genome of *I*. *rubescens* from Lushan, Henan province (*IR-L*) was sequenced, annotated, and analyzed. We found that it has the typical cpDNA quadripartite structure and contains 113 unique genes; four rRNA genes, 30 tRNA genes, and 79 protein-coding genes. Furthermore, the SSRs and sequence repeats identified in the *I*.*rubescens* chloroplast genome can be useful molecular markers to examine genetic diversity and species differentiation in *I*. *rubescens* and related species in future research. In addition, a phylogenetic analysis showed that the chloroplast genomes of the *I*. *rubescens* collections from three different geographical regions did not all group together; one of them was closer to the single accession of *I*. *serra* included in this study. This result revealed that there is intraspecific genetic variation present in *I*. *rubescens*. and implies that there is a regional component to this variation. Above all, our data provides a molecular basis for the further exploration of genetic variation in *Isodon* populations.

## Supporting information

S1 Fig Sequence alignment of chloroplast genomes from three accessions of *I*. *rubescens* by mVISTA. Gray arrows above the alignment indicate the position and direction of transcription of each gene. The scale on the vertical axis shows the percent sequence identity between 50% and 100%. *IR-J*, *IR-X*, and *IR-L* are the three accessions of *I*. *rubescens*.
(TIF)

S1 Table Raw data quality information for the *I*. *rubescens* chloroplast genome. (DOCX)

S2 Table Base composition of the *I*. *rubescens* chloroplast genome. (DOCX)

S3 Table The intron and exon analysis of intron-containing genes in the chloroplast genome of *I*. *rubescens*. (DOCX)

S4 Table Summary of the features of five chloroplast genomes from three species of *Isodon*. (DOCX)

S5 Table SNPs and indels present in the *I*. *rubescens* chloroplast genomes. *IR-J*, *IR-X*, and *IR-L* are the three accessions of *I*. *rubescens*.
(XLS)
