# Supplementary figures and images for: Comparative analysis of chloroplast genomes reveals phylogenetic relationships and intraspecific variation in the medicinal plant Isodon rubescens
Source: PLoS One. 2022 Apr 6;17(4):e0266546. doi: 10.1371/journal.pone.0266546 (PMC8985940; doi:10.1371/journal.pone.0266546)

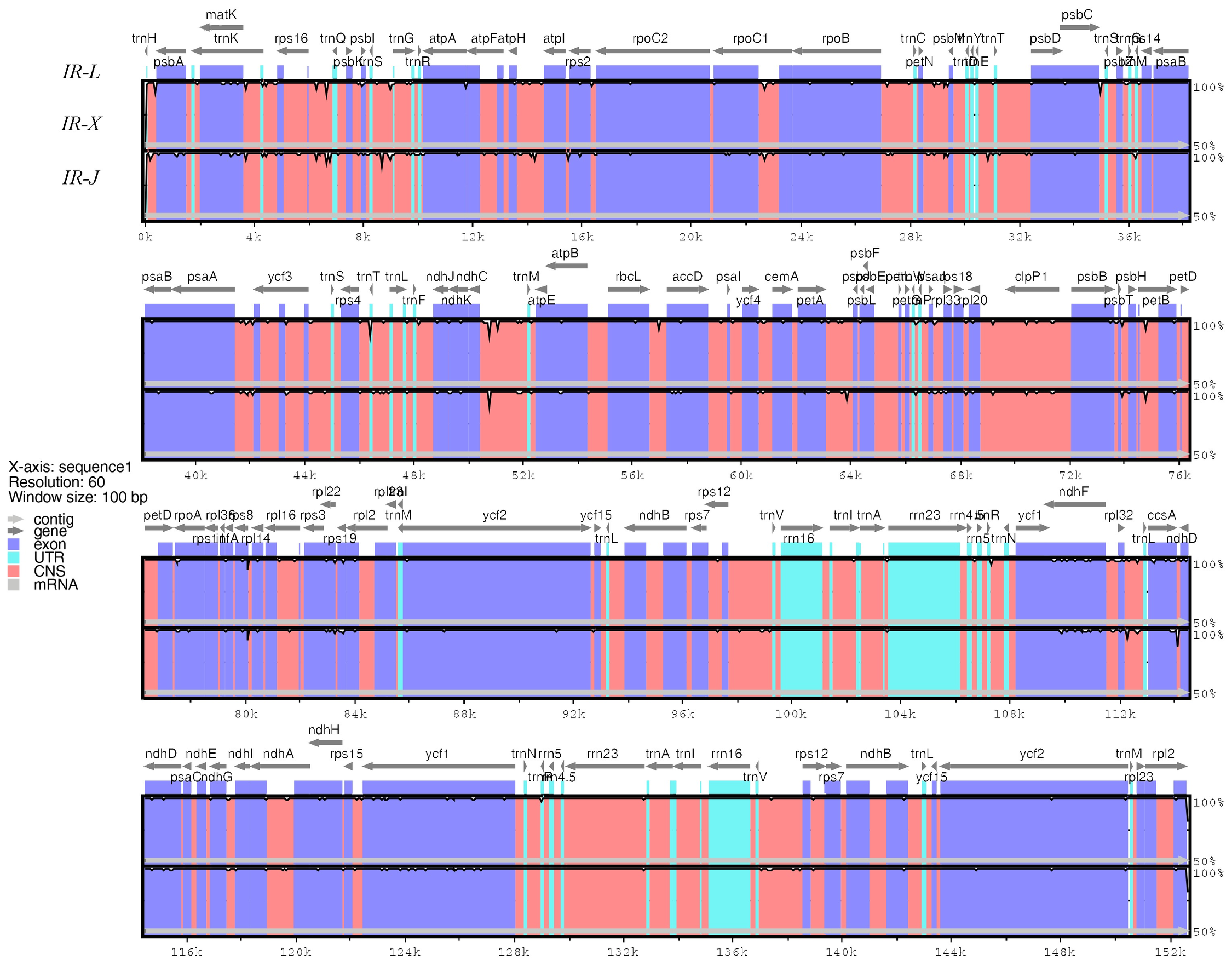

Supplement: S1 Fig — Gray arrows above the alignment indicate the position and direction of transcription of each gene. The scale on the vertical axis shows the percent sequence identity between 50% and 100%. IR-J, IR-X, and IR-L are the three accessions of I. rubescens. (TIF) [file pone.0266546.s001.tif]
