# Supplementary material for: Comparative analysis of chloroplast genomes reveals phylogenetic relationships and intraspecific variation in the medicinal plant Isodon rubescens
Source: PLoS One. 2022 Apr 6;17(4):e0266546. doi: 10.1371/journal.pone.0266546 (PMC8985940; doi:10.1371/journal.pone.0266546)
Supplement: S1 Table — (DOCX) [file pone.0266546.s002.docx]

**S1 Table. Raw data quality information of *I. rubescens* chloroplast genome**

|  | **R1** | **R2** | **ALL** |
| --- | --- | --- | --- |
| **Total Reads Count (#)** | 13,462,561 | 13,462,561 | 26,925,122 |
| **Total Bases Count (bp)** | 1,936,326,598 | 1,968,155,917 | 3,904,482,515 |
| **Average Read Length (bp)** | 143.83 | 146.19 | 145.01 |
| **Q10 Bases Count (bp)** | 1,921,650,308 | 1,959,608,872 | 3,881,259,180 |
| **Q10 Bases Ratio (%)** | 99.24% | 99.57% | 99.41% |
| **Q20 Bases Count (bp)** | 1,874,967,577 | 1,929,387,616 | 3,804,355,193 |
| **Q20 Bases Ratio (%)** | 96.83% | 98.03% | 97.44% |
| **Q30 Bases Count (bp)** | 1,713,820,393 | 1,802,685,214 | 3,516,505,607 |
| **Q30 Bases Ratio (%)** | 88.51% | 91.59% | 90.06% |
| **N Bases Count (bp)** | 1,020,872 | 761,170 | 1,782,042 |
| **N Bases Ratio (%)** | 0.05% | 0.04% | 0.05% |
| **GC Bases Count (bp)** | 701,745,506 | 715,853,291 | 1,417,598,797 |
| **GC Bases Ratio (%)** | 36.24% | 36.37% | 36.31% |
