# Supplementary material for: Comparative analysis of chloroplast genomes reveals phylogenetic relationships and intraspecific variation in the medicinal plant Isodon rubescens
Source: PLoS One. 2022 Apr 6;17(4):e0266546. doi: 10.1371/journal.pone.0266546 (PMC8985940; doi:10.1371/journal.pone.0266546)
Supplement: S2 Table — (DOCX) [file pone.0266546.s003.docx]

**S2 Table. Base composition of the *I. rubescens* chloroplast genome.**

| Region | Length | A (%) | T (U) (%) | C (%) | G (%) | A + T (%) | G + C (%) |
| --- | --- | --- | --- | --- | --- | --- | --- |
| LSC | 83527 | 31.4% | 32.9% | 18.2% | 17.4% | 64.3% | 35.6% |
| SSC | 17663 | 34.6% | 34.4% | 16.2% | 14.8% | 69.0% | 31.0% |
| IR A | 25726 | 28.3% | 28.5% | 22.3% | 20.8% | 56.8% | 43.1% |
| IR B | 25726 | 28.5% | 28.3% | 20.8% | 22.3% | 56.8% | 43.1% |
| Total | 152642 | 30.8% | 31.6% | 19.1% | 18.5% | 62.4% | 37.6% |
