# Supplementary material for: Comparative analysis of chloroplast genomes reveals phylogenetic relationships and intraspecific variation in the medicinal plant Isodon rubescens
Source: PLoS One. 2022 Apr 6;17(4):e0266546. doi: 10.1371/journal.pone.0266546 (PMC8985940; doi:10.1371/journal.pone.0266546)
Supplement: S3 Table — (DOCX) [file pone.0266546.s004.docx]

**S3 Table. The intron and exon analysis of intron contain genes in the** **chloroplast genome of *I. rubescens***

| **No.** | **Gene** | **Location** | **Exon I** | **Intron I** | **Exon II** | **Intron II** | **Exon III** |
| --- | --- | --- | --- | --- | --- | --- | --- |
| **1** | *ndhB* | RA | 777 | 675 | 756 |  |  |
| **2** | *petB* | LSC | 6 | 713 | 642 |  |  |
| **3** | *petD* | LSC | 8 | 710 | 475 |  |  |
| **4** | *rpl2* | RA | 391 | 654 | 434 |  |  |
| **5** | *rps12* | RA | 114 | 804 | 232 | 535 | 26 |
| **6** | *trnA-UGC* | RB | 38 | 806 | 35 |  |  |
| **7** | *trnG-UCC* | LSC | 23 | 689 | 48 |  |  |
| **8** | *trnI-GAU* | RB | 37 | 947 | 35 |  |  |
| **9** | *trnL-UAA* | LSC | 35 | 459 | 50 |  |  |
| **10** | *atpF* | SSC | 145 | 691 | 410 |  |  |
| **11** | *clpP1* | LSR | 71 | 698 | 292 | 631 | 228 |
| **12** | *ndhA* | SSC | 553 | 1032 | 539 |  |  |
| **13** | *ndhB* | RB | 777 | 675 | 756 |  |  |
| **14** | *rpl16* | LSC | 9 | 860 | 399 |  |  |
| **15** | *rpl2* | RB | 391 | 654 | 434 |  |  |
| **16** | *rpoC1* | LSC | 432 | 796 | 1611 |  |  |
| **17** | *rps12* | RB | 114 | 804 | 232 | 535 | 26 |
| **18** | *rps16* | LSC | 40 | 879 | 227 |  |  |
| **19** | *trnA-UGC* | RA | 38 | 806 | 35 |  |  |
| **20** | *trnI-GAU* | RA | 37 | 947 | 35 |  |  |
| **21** | *trnK-UUU* | LSC | 37 | 2504 | 35 |  |  |
| **22** | *trnV-UAC* | LSC | 38 | 583 | 35 |  |  |
| **23** | *ycf3* | LSC | 124 | 711 | 230 | 730 | 153 |
