# Supplementary material for: Comparative analysis of chloroplast genomes reveals phylogenetic relationships and intraspecific variation in the medicinal plant Isodon rubescens
Source: PLoS One. 2022 Apr 6;17(4):e0266546. doi: 10.1371/journal.pone.0266546 (PMC8985940; doi:10.1371/journal.pone.0266546)
Supplement: S4 Table — (DOCX) [file pone.0266546.s005.docx]

**S4 Table. Summary of five chloroplast genomes features of *Isodon* genus**

|  | ***IR-L*** | ***IR-X*** | ***IR-J*** | ***Isodon***  ***serra*** | ***Isodon***  ***lophanthoides*** |
| --- | --- | --- | --- | --- | --- |
| **genome size** | 152,642 | 152,761 | 152,727 | 152,676 | 152,195 |
| **LSC length** | 83,527 | 83,656 | 83,618 | 83,564 | 83,095 |
| **SSC length** | 17,663 | 17,659 | 17,657 | 17,680 | 17,699 |
| **IR length** | 51,452 | 51,446 | 51,452 | 51,432 | 51,401 |
| **Number of genes** | 131 | 129 | 133 | 132 | 132 |
| **Number of protein-coding genes** | 86 | 85 | 88 | 88 | 88 |
| **Number of tRNA genes** | 37 | 36 | 37 | 36 | 36 |
| **Number of rRNA genes** | 8 | 8 | 8 | 8 | 8 |

*IR-L*: *I. rubescens* (MW018469.1) from Lushan, Henan Province. *IR-X*: *I. rubescens* (MW018469.1) from Xianyang, Shaanxi Province. *IR-J*: *I. rubescens* (MW018469.1) from Jiyuan, Henan Province.

*IR-J*, *IR-X*, and *IR-L* are the three accessions of *I. rubescens*.
